# Effect of Endodontic Irrigating Solutions on Radicular Dentine Structure and Matrix Metalloproteinases—A Comprehensive Review

**DOI:** 10.3390/dj10120219

**Published:** 2022-11-23

**Authors:** Abayomi Omokeji Baruwa, Jorge N. R. Martins, Tatjana Maravic, Claudia Mazzitelli, Annalisa Mazzoni, António Ginjeira

**Affiliations:** 1 Department of Endodontics, Faculdade de Medicina Dentária, Universidade de Lisboa, Rua Professora Teresa Ambrósio, 1600-277 Lisboa, Portugal; 2 Grupo de Investigação em Bioquimica e Biologia Oral, Unidade de Investigação em Ciências Orais e Biomédicas (UICOB), 1600-277 Lisboa, Portugal; 3 Centro de Estudo de Medicina Dentária Baseada na Evidência (CEMDBE), 1600-277 Lisboa, Portugal; 4 Department of Biomedical and Neuromotor Sciences, DIBINEM, University of Bologna, Via S. Vitale 59, 40125 Bologna, Italy

**Keywords:** endodontics, irrigating solutions, matrix metalloproteinases, narrative review, radicular dentine

## Abstract

Irrigating solutions play an important role in the eradication of intracanal microbes and debris dissolution during endodontic treatment. Different combinations of solutions and protocols have been advocated, with sodium hypochlorite (NaOCl), ethylenediamine tetra acetic acid (EDTA), and chlorhexidine (CHX) remaining the most widely used ones by many clinicians. Although these solutions provide efficient inorganic dissolution and antimicrobial capacity, their use has also been reported to cause undesired effects on root dentin composition and mechanical and biomechanical properties, such as microhardness, surface roughness, bond strength, and matrix metalloproteinase (MMP) activity. Several corroborating studies attribute these changes in mechanical properties of dentine to the use of irrigating solutions, and there are limited reports on how the solutions affect the expression of MMPs, which may be a correlating link to understanding the role of these enzymes in dentin collagen and changes in the mechanical properties of dentin. Hence, using the basis of several studies from the literature, the objective is to comprehensively review the influence of individual and combined irrigating solutions on root dentine structure and the activity of the MMPs.

## 1. Introduction

The role of microorganisms in root canal system infections and periradicular tissues is unequivocal as the pulp tissue necrosis process progresses, leading to periapical disease [1]. Hence, the prognosis of endodontic treatment is primarily dependent on adequate debridement of the root canal space to reduce and/or eliminate the microbes [2]. This objective is often achieved with the combination of mechanical instrumentation and the use of irrigating solutions [3]. In addition, a successful endodontic treatment subsequently requires an adequate coronal restoration to secure the endodontic seal and to prevent undesirable tooth fractures [4,5].

Notwithstanding the continuous efforts made to comply with these requirements, multiple factors could influence the outcome of the endodontic and restorative treatment at different stages. These may include the histological anatomy of the tooth, the organic and inorganic substrate compositions, the root canal system configuration, the type of microorganisms involved and their byproducts, the chemical agents and protocols employed in the disinfection of the root canal space, the materials and techniques used in sealing and filling the root canal space, and the type of coronal restoration [6,7,8]. Multiple studies, over several years, have explored most of these factors with impactful contributions to the field, which has helped in the current trends of evidence-based dental practices. However, with new clinical protocols and innovations in dental materials there are new associated challenges, which require continuous research studies to provide an adequate scientific basis for offering solutions and enhancing the durability of the restored tooth [8,9,10].

There is evidence that vital teeth are less susceptible to fractures than endodontically treated ones [6,7,8,9,10,11]. In the latter, changes in the chemical and physical properties of dentin after endodontic treatment [12,13,14] would result in crack formation and a weaking of the dental tissue that is now more prone tooth fracture [9,14,15,16].

Ideally, physical characteristics and the chemical composition of dentin should not be adversely affected after the use of an irrigant solution in the root canal and the dentinal tubules [17]. Nevertheless, studies have revealed that changes in the organic and inorganic components of dentin occur with the use of irrigating solutions irrespective of their beneficial properties [18,19,20,21,22,23]. These structural changes may include decrease in bond strength, micro- and nanohardness, and modifications in surface roughness [14,24,25,26].

Furthermore, the structural changes derived using some irrigating solutions could also influence the nature and adhesion of microorganisms as well as the tightness of the seal between the dentin and the endodontic sealers [27,28,29]. The alterations of the interactions between treated dentine surfaces and sealing materials may be due to the softening effect by the irrigating solutions, which allows fast preparation and enables maneuvering of small tight root canals [30]. Reports have also shown that adhesion to the coronal dentin is different from the root dentin and the difficulty of achieving adhesion increases with apical progression in the root canal space [31]. These difficulties have been attributed to changes in the morphology of root dentin, mineral concentrations, fewer dentinal tubules when compared to the coronal dentin, and use of chemical agents during root canal treatment [31,32]. The effect of individual irrigating solutions and the combination of these solutions on the physical properties of dentin have been widely studied. There are still limited reports in the literature that have addressed the effect of the different endodontic irrigation solutions and protocols on the activity of matrix metalloproteinases (MMPs) within the radicular system following endodontic treatment. Within the last two decades, MMPs 2 and 9, which were associated with the degradation of resin–dentin-bonded interfaces in coronal dentin, were also isolated in the demineralized root dentin [33,34,35], and it has been reported that there is an increased activity following the use of resin adhesives [33]. Until now, only two scientific reports by Retana-Lobo et al. [36] in 2021 and Baruwa et al. [37] in 2022 have documented the effect of individual or combined irrigating solutions employed during endodontic treatment on the activity of MMPs in root dentin with the latter study correlating the effect of MMPs with bond strength. However, for this review, emphasis will be placed on NaOCl, CHX, EDTA, and citric acid because they are the most widely used irrigating solutions during root canal treatment.

Hence the aim of this comprehensive review is to elaborate the influence of individual and combined irrigating solutions on root dentin structure and the activities of MMPs within the root dentin.

## 2. Effects of Irrigating Solutions on Dentin Structure

To achieve an effective chemomechanical preparation of the root canal system space, several irrigant solutions have been adopted in root canal treatment, which include, but are not limited to, the following: ethylenediamine tetra acetic acid (EDTA); citric acid (CA); sodium hypochlorite (NaOCl); chlorhexidine (CHX); iodine potassium iodide; hydrogen peroxide; local anesthetic; and saline and/or water [38].

Irrespective of the various irrigating solutions available, a systematic review revealed that there is no difference between these several solutions, and the deficit in well-conducted clinical studies should be taken into consideration when considering a “no difference” result as opposed to taking it for granted [39].

Although irrigating solutions may differ in their actions and chemical properties [40], from the range of characteristics an ideal irrigant should possess, only a few of them, when used alone, offer some spectrum of the ideal properties [39]. Some of these include germicidal, fungicidal, nontoxic, nonirritating, stability in solution, noninterference with tissue repair, prolonged antimicrobial effect, and relatively inexpensive [33,41].

### 2.1. Effect of Sodium Hypochlorite

NaOCl is a broad-spectrum antimicrobial solution used in endodontics to eliminate biofilms of species and microorganisms, such as enterococcus, actinomyces, and candida species [42]. Consequently, it is widely recognized for its effective antibacterial activity, and at minimal concentrations, it destroys bacteria rapidly [3]. Furthermore, it possesses tissue dissolution ability and endotoxin deactivation, and it is nonallergenic [40]. It is also inexpensive, has a long shelf-life, and is easily available [43,44,45]. Regardless of the many advantages of NaOCl, its relative toxicity, inability to remove the smear layer, and unpleasant taste have been condemned [46,47].

It has been recorded that a concentration of NaOCl as high as 10% has been used by dentists [48]. Although an increase in the solution concentration enhances its tissue dissolution effect [49], it could also adversely affect dentin properties [50]. Coupled with an increase in the concentration of hypochlorite solution, other factors that may enhance the tissue dissolution effect of the irrigant include an increase in pH, prolonged exposure time, increase in temperature, and ultrasonic agitation [40,51,52].

As a nonspecific oxidizing agent, the adverse effect of NaOCl is also concentration dependent as with its antimicrobial and tissue dissolving effects [53]. For this reason, it has been utilized for hard tissue deproteination [54]. As an oxidizing agent, its ability to fragment peptide chains and to chlorinate protein terminal groups to produce N-chloramines [55,56] is responsible for its deleterious effects on the dentin surface [25]. Reports have also demonstrated that sodium hypochlorite changes the mechanical properties of dentin by the degradation of the dentin’s organic constituents, and this is because the organic constituent of dentin is 22% of its weight and the irrigant can easily deplete it if dentine is demineralized [25,57].

It was noted from a study carried out on bovine dentin that alterations of the chemical and physical properties occur within the timespan of endodontic treatment [58]. Based on the study of the effect of NaOCl on human root dentin by Marending et al. [25], it was demonstrated that a hypochlorite solution dissolves the organic constituent of dentin but leaves the inorganic constituents unaltered. However, because of the varying methodologies and experimental parameters (i.e., lack of standardization), studies have revealed contradicting results concerning the effect of sodium hypochlorite on the organic constituent of dentine [25].

Compared to a physiological saline solution, the mechanical properties of dentin, such as flexural strength and the modulus of elasticity, have been reported to become remarkably reduced after exposure to a greater than 3% weight per volume of NaOCl for 2 h [8,59]. Additionally, a 2.5% NaOCl solution decreases the flexural strength of dentin after 24 min exposure [26], while with a 5.25% NaOCl solution, both the elastic modulus and flexural strength of dentin decrease after a 2 h exposure [8]. Even a lower 0.5% hypochlorite solution has a comparatively lesser effect on the flexural strength and modulus of the elasticity of dentin when compared to the 6.0% concentration, which is used by several clinicians in the United States of America [8]. The changes in the flexural strength and elastic modulus caused by a high concentration of NaOCl may lead to a decrease in the properties by 50% [25]. On the other hand, a contradicting study by Machnick et al. [60] observed that the flexural strength and elastic modulus were not affected by the sodium hypochlorite concentration. 

Corresponding to the study by Lee et al. [61], who revealed that cracks were on the dentin surfaces after exposure to 5% NaOCl, Marending et al. 2007 [25] also noted crack lines in the dentin specimens after exposure to a 5% hypochlorite solution for 2 h. These results support the hypothesis that the microhardness of root dentin is dependent on the concentration of the hypochlorite used [58]. The microhardness of dentin was depleted after immersion in 1.0% sodium hypochlorite for 15 min [62] and 5.0% hypochlorite solution for 60 s [30], which also corroborates the findings that there is a greater effect on the microhardness when an increased concentration is used.

According to Marending et al. [25], the permeability of dentin is also altered by exposure to NaOCl. The study revealed that dentin permeability was significantly increased after exposure of dentin bars to a 1.0% hypochlorite solution but most especially to a 5.0% concentration. This experiment was carried out by subjecting the dentin specimens treated with NaOCl in a basic fuchsin dye. Changes of the sealing ability and adhesion of the resin-based cement to the dentinal collagen are also consequences of the effect of NaOCl on the dentin collagen [63,64,65]. This effect may produce equal or superior bonding results for some dentin-bonding systems [66,67,68,69,70].

Ari et al. [71] also revealed that 2.5% to 5.25% of NaOCl caused a remarkable increase in the surface roughness, while other data recorded no effect on roughness [14]. A study using the in silico approach observed an increase in stress and strain concentrations with the use of a hypochlorite solution [60,72]. There was a 15.9% increase in tensile strain and a 33.5% increase in compressive strain after a high hypochlorite solution was used as an irrigant [8].

Reports also show that alterations of the dentin matrix are a result of all NaOCl concentrations [71,73]. Zhang et al. [23] reported that a 5.25% hypochlorite solution caused greater dentinal erosion compared to a 1.3% concentration, and the mechanism by which NaOCl removed the dentin organic phase was by infiltration into the apatite-encapsulated collagen matrix. This was possible due to the low molecular weight of the irrigant [23]. Another study on the tissue dissolution and modifications in the dentin composition by different NaOCl concentrations observed that the uninterrupted degeneration of the dentin surface collagen was evident by the time-dependent effect in the increase in the amide II/phosphate ratio [74]. Preceding studies in line with this outcome also proved that the removal of the dentin organic phase was time-dependent [23]. Furthermore, Tartari et al. [74] also confirmed that the degeneration of dentin collagen by a hypochlorite solution was concentration dependent. In this study, both the concentration and time-dependent effects of NaOCl were evident by the decrease in the carbonate/phosphate ratio after 30 s immersion in all concentrations. Apart from the degenerating effects on collagen, another study demonstrated the effect of NAOC on chondroitin sulfate and more specifically on type I collagen, while others demonstrated the loss of immunoreactivity of both glycosaminoglycans and type I collagen after treatment with sodium hypochlorite [75]. 

The consequences of damage to the collagen matrix of dentine included a less resilient and a more fragile substrate [25,76], which may encourage the generation of fatigue cracks when cyclic forces are applied and ultimately cause a decrease in the resistance to crown and root fractures [23,77]. The deproteination ability of the hypochlorite solution led to an unequal effect thus producing an unbound hydroxyapatite and collagen-sparse dentine subsurface rich in apatite [76,78]. The deproteination effect also caused additional dentin changes evident by the “moth-eaten” appearance seen on a scanning electron microscope (SEM) [66,79]. The microscopy analysis revealed undamaged intertubular dentin surface as effects of NaOCl on the dental tissue [80].

In as much as there have been insufficient studies on the direct quantitative effects of low concentrations (0.5–2.25%) and short exposure periods (1–10 min) of NaOCl on dentin deproteination, 0.5% sodium hypochlorite causes a lesser effect on dentin deproteination than 1.0% and 2.25% concentrations [81]. Deproteination by 5.0% NaOCl for 10 min decreased the bond strength between the fiber posts and dentin surfaces [82].

To maintain an aseptic environment in the root canal while trying to limit dentin deproteination, it is advised that an ideal NaOCl concentration at a suitable exposure time is used [76]. Hence the use of hypochlorite concentrations, such as 1.0% and 2.5%, which have been found to promote organic tissue dissolution as well as limited destruction to the dentin structure, have been advised [74]. Although preceding studies revealed that 0.5% NaOCl is effective only for the removal of bacteria on the dentin surface layer, other studies recommended the use of a low 0.5% hypochlorite solution concentration as a routine irrigating solution but at a longer exposure period because it achieved excellent antimicrobial activity and had an insignificant effect on dentin deproteination [14].

### 2.2. Effect of Decalcifying Agents

The various levels of actions of decalcifying agents on mineral dentin depend on the concentration, immersion time, and decalcifying capability [74]. Irrespective of the concentration and time, EDTA and other chelating agents can cause harmful effects. Exposure to 17% EDTA for 3 min can dissolve the inorganic smear layer of dentin, and because EDTA has been found to remove the smear layer [14], other chelating agents, such as etidronic acid (HEDP), tetrasodium ethylenediamine tetraacetic acid (EDTANa_4_), and peracetic acid (PAA), have also been used as substitutes of EDTA [74] to remove the smear layer. It has been noted that in 5 min, HEDP and EDTANa_4_ completed this action [28,83], while in 1 min, PAA could remove the inorganic components of the smear layer [84].

The study by Tartari and Bachmann [74] revealed that changes in the components of dentin were evident by the ratios of amide III/phosphate (PO_4_^3−^ v3) and carbonate (CO_3_^2−^ v2)/phosphate (PO_4_^3−^ v3) bands. At different rates, increments in the amide II/phosphate ratio indicated dentin demineralization and dentin surfaces sparse in apatite but rich in collagen, while a decrease in the phosphate and carbonate apatite bands by the different decalcifying agents at different rates was also observed. At 9% and 18%, HEDP did not change the amide III/phosphate ratio; however, at 5% and 10%, EDTANa_4_ minimally reduced the phosphate group compared to the other decalcifying agents. At a 2% concentration, PAA eliminated the phosphate group and exposed the collagen matrix, which ultimately caused a significant increase in the amide III/phosphate ratio. The results by Tartari and Bachmann [74] reveal that HEDP and alkaline EDTANa_4_ have little effect on dentin demineralization, while EDTAHNa_3_ and PAA, at concentrations greater than 0.5%, may have a greater demineralizing ability and should be prudently used [21].

The process by which EDTA removes calcium ions (Ca^2+^) from mineral tissues is shown to destroy the dentin matrix [17], and it has been noted that calcium was depleted from the dentin surface to a depth of almost 150μm after exposure to 17% EDTA for 2 h [85]. Although EDTA destroys the dentin matrix [17], its solo use as an irrigant could inhibit dentin dissolution because of the accumulation of an organic matrix on the canal surface [75].

EDTA has been found to be a major cause of dentinal erosion [86], and because the erosion of the canal wall is a result of prolonged used of the solution due to its ability to demineralize root dentin [87,88], efforts have been made to weaken the constant chelating effect of EDTA for longer periods [89,90]. The use of a lower EDTA concentration (1%) was recommended by Şen et al. [90] to prevent extreme root canal dentin erosion but also to provide sufficient smear layer removal. Apart from EDTA’s effect on the dentinal wall, 19% citric acid has also been reported to enlarge the superficial part of dentinal tubules [91,92].

Although the bond strength between endodontic sealers, such as AH plus (Dentsply DeTrey, Konstanz, Germany), and dentin is enhanced due to the chemical bond between the material and the amino groups of dentin that is exposed by the demineralizing effect of strong decalcifying agents (EDTAHNa_3_ & PAA) [65], the generation of a weak bond and an increase in interfacial degradation could occur due to insignificant penetration of the material into the demineralized dentin caused by significant decalcification in the walls of the root canal [28,93,94,95,96].

Additionally, deleterious effects on dentin’s surface roughness provoked by EDTA exposure has been reported [8,24]. Ari et al. in 2004 [71] also reported a substantial increase in the surface roughness on root canal dentine by 17% EDTA. Other dentin properties, such as micro and nanohardness, have also been reported to be changed using chelating agents [14], and this corroborates the studies by Saleh et al. in 1999 [30] and Cruz-Filho et al. in 2001 [97] that demonstrated a decrease in dentin’s microhardness by EDTA.

Moreover, the teeth fracture resistance was found to be markedly reduced by 17% EDTA when compared to 5% EDTA after a 10 min exposure, even though after a 1 min immersion, it was reported that 5% EDTA had a greater effect on the reduction of teeth fracture resistance when compared to 17% EDTA [98]. Additionally, demonstrations by Uzunoglu et al. [98] revealed that when compared to roots rinsed with distilled water only, 17% or 5% EDTA produced a higher fracture resistance of the roots after 1 min exposure, which was explained by the ability to remove the smear layer, and this has been shown to improve the bond strength of resin-based sealers to dentin [98].

Following dentin exposure to 17% EDTA for 2 h, the flexural strength and the modulus of elasticity were reduced by one third and by half, respectively [99]. Because mechanical properties, especially strength, are less likely to be affected after the use of EDTA for shorter periods, it is recommended to reduce the influence of high-concentration EDTA (15% or 17%) for it to be used during shorter periods (approximately 2 min) [98,100,101,102,103,104,105].

### 2.3. Effect of Chlorhexidine

Chlorhexidine (CHX), a cationic biguanide with a broad spectrum, is the most used antiseptic product [3]. It is used as a disinfectant, preservative, and antiseptic in the medical, pharmaceutical, and dental fields [106,107]. The bis-biguanide is a strong basic salt, and in its original form (chlorhexidine acetate and hydrochloride), it is most stable, but due to its insolubility in water [40,108], chlorhexidine digluconate became its replacement [109]. 

Based on the effect of CHX on the mechanical properties of dentin, only one study proved there was no effect [105]. The association of chlorhexidine molecules with the surface of dentin was responsible for the activity of CHX [110]. The removal of the smear layer by this interaction also increased chlorhexidine activity [111,112].

Ari et al. [71] observed that the microhardness of dentin was markedly decreased by all irrigating solutions (NaOCl, H_2_O_2_, EDTA, etc.) except CHX. They noted that the surface roughness of dentin was not affected by 0.2% CHX gluconate, and their results also reveal that this solution was the only one, amongst the other irrigating solutions, that did not affect the constituents of dentin. Due to its nontoxic effect, no negative effect on microhardness, and roughness of dentin, it was suggested that 0.2% CHX gluconate was a suitable irrigant to be used in endodontics [71]. Further, chlorhexidine did not influence the fracture resistance of endodontically treated teeth after irrigation, while insufficient smear layer removal using chelating agents may inhibited its beneficial effects [98]. It has also been noted that the demineralization of dentin and exposure of dentinal tubules by 17% EDTA reinforced the effect of chlorhexidine [113].

Compared to other irrigating solutions, CHX showed the greatest bond strength values [114]; by reducing the wetting angle and raising the surface energy, it improved the penetration and bond strength of AH Plus (Dentsply, Petrópolis, Rio de Janeiro, Brazil) and wettability of dentin [115]. Worthy of note is the fact that CHX can prevent the destruction of collagen by host-derived proteases MMPs [95,116].

## 3. Effect of Combined Irrigating Solutions on Dentin

The idea of combining irrigating solutions is to tackle some drawbacks when the procedure is performed with a unique chemical agent [117,118]. The combination of demineralizing agents with a NaOCl solution did not cause a temporary loss of the required properties of the solutions [83,117,119]. However, the combination of NaOCl with demineralizing agents could result in a dentine collagen exposure enhancing restoration of the dentin organic/inorganic natural composition for adhesion with dental materials (endodontic sealers and resin cements) [83].

Up until now, there has been no agreement on the best irrigation sequence [120], even though previous studies revealed that to remove the smear layer effectively, 10 mL of 5.25% NaOCl should be used after a 10 mL of 17% EDTA [121]. The alternate application of EDTA and NaOCl to an uninstrumented root canal dentin resulted in an enlargement of the tubular orifice diameters and eroded the appearance [22].

Qian et al. [17] observed that the use of 17% EDTA or 10% citric acid or 17% ethyleneglycoltetraacetic acid (EGTA) for 1 min as the first rinse followed by 5.25% NaOCl resulted in eroded peritubular and intertubular dentin as well as in enlarged and rough dentinal tubular orifices. However, exposure to 5.25% NaOCl as the initial rinse followed by the decalcifying agents resulted in regular dentinal tubular orifices and a smooth dense intertubular dentin surface [17]. These results occurred because the decalcifying agents (EDTA/citric acid) used as the initial rinse exposed the collagen fibers, enabling the hypochlorite solution used as the final rinse to directly destroy the collagen, whereas NaOCl as the first rinse cannot destroy the collagen because of the protection of the hydroxyapatite coating [17]. Hence, the ability of EDTA to decalcify inorganic components, and the ability of NaOCl to dissolve organic matrix have been advocated [87].

Preceding studies noted a substantial increase in erosion when the exposure periods were prolonged for the demineralizing agents used first and NaOCl solution used last [87]. Hence, the use of 17% EDTA for not more than 1 min has been proposed [122]. In the study by Kaya et al. [80], who used EDTA at a constant concentration and application time but changed the concentration of NaOCl, noted that a 5.25% hypochlorite solution was the causative factor of dentinal erosion. This result brought about the hypothesis that the erosive effects of the combination of NaOCl and EDTA are independent of EDTA. This hypothesis corroborates the preceding observations by Zhang et al. [23]. The summary of the effect of irrigating solutions on dentine is highlighted in Table 1.

The use of combination solutions resulted in surfaces having various chemical functional groups that enable several types of adhesion with dental materials, and there was a direct relationship between the functional groups and the solution used as a final rinse [74]. Although the mechanism by which irrigating solutions affect the bond strength to dentine is not fully elucidated, the infiltration of resin-bonding materials is affected by the changes in the collagen and prostaglandin caused by oxidant radicals [75]. Prado et al. [115] noted a higher bonding ability when phosphoric acid and hypochlorite solutions were combined or when EDTA was combined with CHX, while Nassar et al. [123] reported a substantial increase in the bond strength value of an epiphany root canal sealer to dentine when 5% NaOCl was used as the initial rinse followed by 2% CHX or 10% sodium ascorbate solutions. The bonding ability of materials to dentin was also adversely affected by the combination of 3% hydrogen peroxide (H_2_O_2_) and 5% NaOCl [75]. Neelakantan et al. [65] proposed that to decrease the adverse effects of NaOCl on the bond strength of an epoxy resin-based root canal sealer, EDTA should be used afterward.

Varying results on the effect of NaOCl and EDTA on the modulus of elasticity have been reported. There were deleterious effects on the elastic modulus when 2.5% NaOCl and EDTA were used for a 2 h exposure time [59,60], while in another study, after a 3 min exposure, there was an absence of effect on the modulus of elasticity of the dentin [124].

The effects of alternating irrigating solutions on dentin hardness can be attributed to the dissolution properties of the hypochlorite solution on the organic components (collagen) of dentin [71,125,126]. An EDTA/NaOCl combination used as an initial rinse followed by CHX intensified the fracture resistance of the roots filled with AH plus (Dentsply DeTrey, Konstanz, Germany) [120]. The fracture resistance of the teeth treated with 17% EDTA plus 2.5% NaOCl was found to be lower when compared to the teeth treated with 5% EDTA plus 2.5% NaOCl; however, when compared with CHX, the fracture resistance increased after the specimens were treated with both 17% and 5% EDTA. The exposure of the dentinal tubules coupled with the increased dentinal demineralization by the 17% EDTA was found to be responsible for the exceptional strengthening effect of CHX [120].

When EDTA was used alone as an irrigating solution, there was a limitation of dentin dissolution because of the accumulation of the organic matrix on the canal surface [75]. However, when NaOCl was used after EDTA, it enhanced the removal of the organic matrix thereby exposing the inorganic components [19,93]. In addition, a remarkable elimination of calcium ions with the combined use of 17% EDTA and 2.5% NaOCl for 1 mi was noted when compared to the 17% EDTA used alone [127]. When the solutions were combined, the dentine deproteination facilitated by NaOCl was evident by the changes in the amide III/phosphate [23,74,76,81,128,129]. It was demonstrated by Tartari et al. [74] that there was an increase in the amide II/phosphate ratio when demineralizing agents were used after the hypochlorite solution. Moreover, they noted that HEDP caused the least increase in the amide/phosphate ratio followed by EDTAHNa_3_, while PAA caused the greatest increase. 

However, because none of these solutions could meet all the requirements of irrigation in endodontics, a combination of these solutions and regimens has been advocated, and several studies have inclusively reported it to be successful at reducing the microorganism load within the root canal space [3,130,131].

## 4. Effect of Irrigating Solutions on Endogenous Matrix Metalloproteinases (MMPs)

MMPs are calcium-dependent, zinc-containing, cell-secreting proteolytic enzymes, which were first described in 1962 by Jerome Goss and Charles Lupiere in tadpole tail metamorphosis [132]. Numerous studies have identified over 20 MMPs in humans, [133,134] and they have been classified and grouped according to their cellular localization and substrate specificity as collagenases (Types 1, 8, 13, and 18), gelatinases (Types 2, 9), stromelysins (Types 3, 10, and 11), matrilysins (Types 7 and 26), membrane-type MMPs (Types 14–17, 24, and 25), and others (Types 12, 19, 20–23, 27, and 28) [134].

In dentistry, several MMPs (Types 2, 3, 7, 8, 9, and 20) have been identified by multiple studies in human coronal and root dentine, pulp tissue, saliva, and carious lesions [135,136,137,138,139,140,141,142], and the role of gelatinases (Types 2 and 9) in dentin–collagen hybrid bond layer degradation has been broadly studied [143,144,145,146]. These studies documented that the MMPs were released locally during caries destruction of dentin and pulp inflammation and in low pH conditions, such as the use of acids during bonding procedures [94,136,138,147]. As the dentin protein matrix is composed of 90% collagen (primarily type I with some amount of type V collagen) and 10% noncollagen protein (dentin matrix protein I, dentine phosphophoryn, and dentin sialoprotein), the gelatinases are known to degrade the type I collagen matrix, which makes them play a major role in adhesive dentistry [140,148].

A previous study by Santos et al. [34] identified 68/72 kDa and 92 kDa bands in root dentin, which corresponded to MMP 2 and −9, respectively, and it has been reported that MMP 2 was detected in all dentin compartments, while MMP 9 had specific distributions, which required an extensive demineralization procedure for its identification with EDTA. Additionally, with the use of citric acid and acetic acid, more clear bands of MMP 9 were seen, which led to the suggestion that EDTA may inhibit its activity [34,146]. 

Until now, two immunohistochemistry studies with corroborating results have reported the influence of individual or combined endodontic irrigating solutions on the activity of MMPs [36,37] on the root dentin (Table 2). Both studies were able to show that the commonly used irrigating solutions (NaOCl, EDTA, citric acid, and CHX) changed the expression of the MMPs. Additionally, both studies also showed a reduced activity of these enzymes when NaOCl was used, although this could be seen as a beneficial effect in maintaining the bond quality to root dentin over time. However, the effect of the solution was reported to be detrimental to dentin due to the dissolution of organic dentin structures by infiltrating into the apatite-encapsulated collagen matrix [23]. When chelating agents, such as EDTA or citric acid, were used, alone or in combination with NaOCl, there was an increase in the activity of the MMPs, which corroborates the findings of several studies on coronal dentine [149], and this was attributed to the demineralizing effect of the chelating agents, which caused the exposure of dentinal proteins, collagen fibers, and activation of latent endogenous MMPs [33,149,150,151].

**Figure 1:**
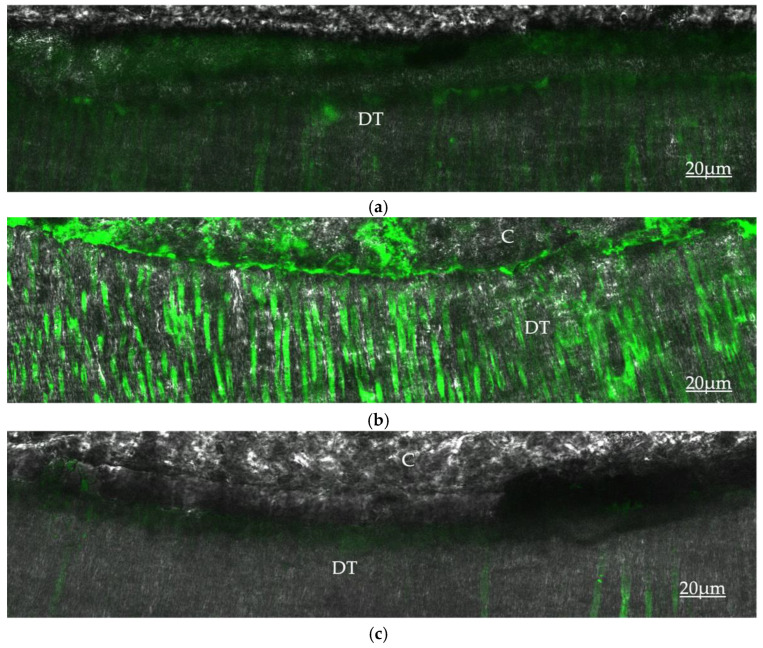
Multiphoton confocal microscopy in situ zymography images showing hydrolyzation activity of fluorescein-conjugated gelatin by the MMPs in dentine following the use of different irrigating solutions: (**a**) 5.25% Sodium hypochlorite; (**b**) 5.25% Sodium hypochlorite and 10% citric acid; (**c**) 5.25% Sodium hypochlorite, 10% citric acid, and 0.2% chlorhexidine. [DT: dentinal tubules; C: resin cement—Core X flow (Dentsply Sirona, Ballaigues, Switzerland)].

However, when chlorhexidine was used as a final rinse, both studies reported a lower expression of MMPs, which corroborated the established finding that chlorhexidine inhibits the activities of MMPs. The in situ zymography images from the latter study [37] (Figure 1a–c) show the varying fluorescence intensity that is emitted by the hydrolyzation of fluorescein-conjugated gelatin by the MMPs in the hybrid layer and dentinal tubules using different irrigating solutions with the highest activity seen with the combination of NaOCl + CA (Figure 1b) and the least activity seen when CHX was used as the final solution (Figure 1c). As the majority of the adhesive protocols in dentistry require the use of an acid in achieving adhesion, which invariably activates the MMPs that degrade the hybrid layer leading to bond failure, several studies have researched the agents that could inhibit the activation of the MMPs, which include chlorhexidine, gallardin [144], and 1-ethyl-3-(3-dimethylamino-propyl) carbodiimide (EDC) [152,153,154], and recently a review study by Amin et al. [155] reported the use of quaternary ammonium compounds, bisphosphonates, and tetracycline. The application of 2% chlorhexidine after acid etching has been extensively researched, and it has been shown to inhibit the expression of MMPs [36,37,153,154], stabilize the adhesive interface, and prevent bond failure [144,145,147].

## 5. Final Remarks and Future Research Directions

Over the years, studies have focused mainly on assessing the influence of endodontic irrigating solutions and chemical agents used in adhesive dentistry on the chemical composition and mechanical properties of the coronal and root dentin. Today, there are well-supported findings that these chemicals or solutions change the dentin structure, which ultimately affects the ability to bond to dentin. With these known facts, there are still some doubts and controversies in the literature regarding the distinct correlation of events that leads to the alteration of the dentine structure and bonding quality, which consists simultaneously of a gap and limitation of the current literature and present review; however, a future lead that could correlate these facts may be hidden in the research of the expression of MMPs in root dentine. Hence, prospective innovative studies that investigate the role of endodontic irrigating solutions on dentin structure and composition with correlation with bond strength would give more clarity to the existing findings.

## Figures and Tables

**Table 1:** Summary of each endodontic irrigating solutions and their reported effects on the physical and mechanical properties of dentine structure.

	Irrigating Solutions		
	NaOCl	EDTA	CHX
**General considerations**			
pH range	11–13	7–9	5–7
Concentrations	0.5–10%	1–17%	0.12–2%
Biocompatibility	Low	Low	High
Mode of action	Bactericidal	Bacteriostatic	Bacteriostatic
Substantivity	Low	Low	High
Tissue dissolution	High **	Low	Low
Smear layer removal	Low	High	Low
**Effect on dentin’s composition**			
Collagen degradation	Medium **	High ^+^	Low
Carbonate/phosphate ratio	Decrease *	Decrease	Low
**Effect on dentin’s physical and mechanical properties**			
Microhardness	Reduce	Reduce	No effect
Erosion	High ^++^	High ^++^	No effect
Surface roughness	Increase	Increase	No effect
Bond strength	Reduce	Reduce	Increase

* Concentration-dependent; ** concentration- and time-dependent; ^+^ time-dependent; ^++^ in combination with other irrigating solution.

**Table 2:** Most relevant methodological aspects and relevant findings on studies addressing the MMP activity after root dentin exposition to irrigating solution.

	Retana-Lobo et al., 2021 [36]	Baruwa et al., 2022 [37]
**Methodological aspects**		
Methodology for assessing MMP activity	Gelatin zymography of dentine powder.	In situ zymography of the hybrid layer.
Irrigating solutions used	Distilled water, 5% NaOCl, 18% EDTA, 2% CHX	Saline, 5.25% NaOCl, 10% Citric acid, 0.2% CHX
Sample size	20 single roots (*n* = 5)	24 single roots (*n* = 6)
**Most relevant findings**		
NaOCl effect on MMPs	Reduced	Reduced
Combination of NaOCl + EDTA/CA effect on MMPs	Increased	Increased
NaOCl + EDTA/CA + CHX effect on MMPs	Reduced	Reduced
Correlation of MMP activity with root bond strength	N/A	Yes

## Data Availability

Not applicable.
